# A Rhesus Monkey Model of Non-suicidal Self-Injury

**DOI:** 10.3389/fnbeh.2021.674127

**Published:** 2021-08-05

**Authors:** Melinda A. Novak, Jerrold S. Meyer

**Affiliations:** Department of Psychological and Brain Sciences, University of Massachusetts, Amherst, MA, United States

**Keywords:** self-injurious behavior, non-suicidal self-injury, rhesus monkey, hypothalamic- pituitary-adrenocortical axis, early life stress, opioid system, sleep disruption

## Abstract

Non-suicidal self-injury (NSSI) is a type of behavioral pathology seen not only in a variety of clinical conditions but also among non-clinical populations, particularly adolescents and young adults. With the exception of rare genetic conditions that give rise to self-harming behaviors, the etiology of NSSI and the events that trigger specific episodes of this behavior remain poorly understood. This review presents the features of an important, extensively studied animal model of NSSI, namely spontaneously occurring self-injurious behavior (SIB) in rhesus macaque monkeys. We compare and contrast rhesus monkey SIB with NSSI with respect to form, prevalence rates, environmental and biological risk factors, behavioral correlates, proposed functions, and treatment modalities. Many parallels between rhesus monkey SIB and NSSI are demonstrated, which supports the validity of this animal model across several domains. Determining the etiology of spontaneously occurring SIB in monkeys, its underlying biological mechanisms, and which pharmacological agents are most effective for treating the disorder may aid in identifying potential risk factors for the occurrence of NSSI in humans and developing medications for severe cases that are resistant to conventional psychotherapeutic approaches.

## Introduction

The objective of this review is to examine the applicability of a rhesus monkey model of self-injurious behavior (SIB) for non-suicidal self-injury (NSSI) in humans. Other models based on pharmacological or genetic manipulations of laboratory rats and mice have been instrumental in studying NSSI associated with genetic syndromes such as Prader-Willi and Lesch-Nyhan, as well as neurodevelopmental disorders like autism (Knapp and Breese, 2016; Devine, 2019). Indeed, these models have pointed to a possible role for the dopamine system in mediating the self-injury observed in those syndromes and disorders. In contrast, the animal model reviewed here differs from experimental rodent models in three important ways: (1) the model is based on a higher primate species that possesses communicative, social, and cognitive abilities more similar to humans than those of rodents; (2) SIB in our model arises spontaneously, without the need for pharmacological treatments; and (3) the model is particularly directed toward understanding NSSI that occurs in the general population and in certain psychiatric disorders such as posttraumatic stress disorder.

The rationale for evaluating the rhesus monkey model of SIB is based, in part, on disturbing trends showing that NSSI is on the rise among teenagers and young adults (Wester et al., 2018) and on significant animal welfare concerns for non-human primates with this condition (Novak, 2020). Humans and Old World monkeys such as rhesus macaques share many important features including a long lifespan, complex social structures, higher order cognitive processes, large brains in relation to body size, and hands adapted for manipulation of objects. From infancy to adulthood, monkeys pass through key developmental stages similar to those in humans. Additionally, Old World monkey brains contain all the major prefrontal cortical subdivisions seen in the human brain (Carmichael and Price, 1994). These similarities suggest that animal models based on these species can serve as an excellent platform to identify potential risk factors, test functional hypotheses, and assess the efficacy of various treatments for NSSI in humans.

Spontaneously occurring SIB has been observed in several species of small mammals (Vergneau-Grosset and Ruel, 2021) and more importantly for the present review, it also occurs in a small percentage of non-human primates living in diverse settings (e.g., research facilities, zoological parks, and natural habitats). Because non-human primate SIB is not experimentally induced, considerable efforts are required to identify the risk factors that lead to this condition. In that sense, these efforts parallel those of clinicians and scientists in characterizing risk and vulnerability in individuals with NSSI. Although spontaneously occurring SIB has been observed in several species of Old World monkeys and great apes, we focus here on a model of this behavioral pathology in rhesus monkeys that has been studied extensively. Below we first identify concordances of rhesus monkey SIB with human NSSI across a number of domains that include form, prevalence, associated features, etiology, and function. We then discuss the value of this animal model for testing potential treatments.

The timing of this evaluation is significant inasmuch as NSSI is under consideration as a distinct disorder (NSSID) in the Diagnostic and Statistical Manual of Mental Health Disorders (DSM-5). However, this proposed classification is not without controversy. Some have argued that NSSI is merely a symptom of other mental conditions, particularly borderline personality disorder and therefore, NSSID has limited value as a distinct diagnostic category (for example, see Ghinea et al., 2020). Others note that NSSI is associated with many different personality disorders and more importantly, it can also occur in non-clinical populations. These arguments support the development of NSSID as a new diagnostic category (for example, see Hooley et al., 2020). This controversy highlights the heterogeneous nature of NSSI, a condition with many potential causes, risk factors, associations, and comorbidities. It would be unreasonable to expect that SIB in monkeys would serve as a model for NSSI broadly. Accordingly, in the present article, we primarily limited the scope of our comparison to the occurrence of NSSI in stress-impacted disorders (especially post-traumatic stress disorder [PTSD]) and in community samples composed of teenagers and young adults with no formal mental health diagnosis. Furthermore, we included information on NSSI in these restricted populations, only as it related to the features present in monkeys with SIB. For some of these features (e.g., hypothalamic pituitary adrenocortical [HPA] axis activity, sleep disruption, and aggressive behavior), the extant literature was small and comprehensive coverage was possible. For other features (e.g., early life stress), the extant literature was so large that comprehensive coverage was not possible. Instead, we relied on recent meta-analyses or systematic reviews. For other aspects such as prevalence rates, the extant literature was so variable, we only attempted to cover the range. Relevant search terms included with NSSI and deliberate self-harm were early life stress, adolescent stress, peer relationships, HPA axis, sleep disruption, insomnia, genetic polymorphisms, genome-wide association studies (GWAS), serotonin, serotonergic, GABAergic, adrenergic, opioids, prevalence, function, and etiology. All relevant papers published prior to February 2021 were examined.

## Challenges to the Study of Self-Injury in Humans and Monkeys

Understanding why organisms engage in self-injury is a complicated endeavor, especially when the early patterns and trajectories of this behavior can be discerned or hypothesized only through retrospective analysis of past history and, in the case of humans, using verbal accounts. Two additional issues come into play in any discussion of the etiology of SIB and NSSI. These include both the presence of wide individual differences among monkeys who develop SIB and humans who show a pattern of NSSI, and equifinality, the idea that a given end state, such as self-injury, can be reached through many different pathways.

### Individual Differences

Rhesus monkeys housed in research facilities typically differ in their genetic background (Driscoll et al., 2016), temperament (Altschul et al., 2016; Bliss-Moreau and Moadab, 2016), clinical health history (Urvater et al., 2000; Wu et al., 2020), and previous experiences (Dettmer et al., 2012; Morin et al., 2019). As we describe below, some of these individual differences conferred vulnerability to SIB and influenced treatment success. Thus, SIB could not be resolved effectively in all monkeys with a standard treatment regimen. In a similar vein, both the presence and severity of NSSI covary with many different factors such as temperament (Buelens et al., 2020) and early life stress exposure (Cassels et al., 2018).

### Equifinality

It is clear from our research that not all monkeys are exposed to the same set of experiences prior to the emergence of SIB. Even if some animals have similar exposures, these experiences may not occur at the same time points. Furthermore, individuals develop at different rates and may not be at the same maturational stage for these variables to induce an effect. This variation in exposure and maturation helps explain not only why some monkeys develop SIB but others do not; it also underscores the principle of equifinality because multiple pathways can lead to the same outcome of SIB. Three diverse examples illustrate this point. First, in the monkeys that exemplify our model, SIB appeared to result from an interaction between genetic factors, early life stress exposure, and a dysregulation of the stress response system (Novak, 2003; Tiefenbacher S. et al., 2005). Second, SIB developed in some monkeys as a consequence of a clinical disease, reactive arthritis (Urvater et al., 2000). Lastly, a case report identified inadvertent reinforcement by animal care staff for increased attention as the source of SIB in an individual baboon (Dorey et al., 2009).

This concept of equifinality also applies to NSSI, which is associated to a greater or lesser degree with many diverse psychiatric disorders and also occurs in non-clinical populations. Although previously identified as a defining characteristic of borderline personality disorder (Stead et al., 2019), NSSI is also linked to other mental health conditions that include, but are not limited to, depressive disorders (Kaess et al., 2012), autism spectrum disorder (Flowers et al., 2020), substance abuse disorders (Escelsior et al., 2021), eating disorders (Pérez et al., 2018), developmental disabilities (Hoch et al., 2016), and posttraumatic stress disorder (Kimbrel et al., 2016). Not all individuals with these disorders also engage in NSSI. However, comparisons of those that exhibit NSSI vs. those that don’t can aid in the identification of the other factors associated with the development of self-injury. For example, individuals with PTSD and a recent history of NSSI were much more likely to make violent threats and act aggressively toward others than PTSD patients without NSSI (Calhoun et al., 2017). In a similar vein, a comparison of two depressed groups – those with NSSI vs. those without – revealed a dysregulation of the HPA axis and elevated perceptions of stress in the NSSI group (Klimes-Dougan et al., 2019). It is unlikely that any animal model, including the one discussed here, could be applicable to all the disorders describe above. Thus, the focus of the present rhesus monkey model as noted above will be on its applicability to the occurrence of NSSI in stress-related disorders, such as PTSD and in non-clinical populations (i.e., community samples of adolescents, college students, military veterans, and other groups that neither have a developmental disability nor have received a formal diagnosis of and/or are under treatment for an anxiety, mood, or psychotic disorder).

## Characteristics of Self-Injury in Human and Monkeys

### Definition and Forms

#### Human Self-Injury

Non-suicidal self-injury is commonly defined as deliberate body-directed mutilation without suicidal intent that is distinct from socially approved forms of harm such as tattoos or piercings (Nock, 2009). However, studies have also shown a strong connection between NSSI and suicidal thoughts and behaviors, indicating that the presence of NSSI may be a precursor to suicide attempts in some individuals (Whitlock et al., 2013). NSSI can take many different forms including cutting, burning, head banging, biting, hitting/punching, and excoriation of wounds (Klonsky, 2011; Kimbrel et al., 2017; Mann et al., 2020). It is not uncommon for individuals to use more than one method, and the more serious methods may require emergency department visits (Ammmerman et al., 2019).

#### Monkey Self-Injury

Self-injurious behavior in non-human primates is defined as any self-directed behavior to the body that causes tissue damage in the form of abrasions, cuts, gashes, and punctures of the skin. We use SIB as the descriptor because suicidal intent cannot be inferred from monkey behavior. The most common form of SIB is biting directed to arms and legs and occasionally to the torso. Because of marked sexual dimorphism in the size of the canine teeth in Old World monkeys, males can produce more serious wounds, such as punctures and gashes, than females. Serious wounds require veterinary treatment. Other forms of SIB include hitting, head banging, and disruption of wound healing (Novak, 2003; Taniguchi and Matsumoto-Oda, 2018). It is uncommon for individuals to use multiple methods, except in the case of excessive manipulation of bite wounds that significantly delays healing. However, monkeys may show a preference for particular body sites, leading to permanent skin damage. Although SIB has been studied most extensively in primate research facilities, it also occurs in primates housed in zoological gardens (Hosey and Skyner, 2007) and those living in natural environments (Grewal, 1981; Taniguchi and Matsumoto-Oda, 2018).

### Prevalence Rates

#### Human Self-Injury

The reported prevalence of NSSI, even within specified non-clinical populations, is highly variable and is likely influenced by the methodology used to collect the data (Muehlenkamp et al., 2012). Researchers generally focus on both lifetime prevalence and the current 12-month prevalence. For example, a review and meta-analysis of NSSI in non-clinical populations estimated a lifetime prevalence of 20% in college students (Swannell et al., 2014). A cross-sectional study of first-year students conducted in 2008, 2011, and 2015 found a dramatic increase in lifetime NSSI prevalence from 20 to 45% across the three cohorts (Wester et al., 2018), suggesting that NSSI has become a growing public health concern. However, prevalence rates of NSSI are typically based on self-report and do not take severity or the need for treatment into account. In a sample of 4,565 first-year university students, lifetime prevalence rates were high (nearly one in five individuals), consistent with other findings. The current year prevalence was 9.5%. However, when *DSM-5* criteria, which take into account the severity of the condition, were applied, current year prevalence rates dropped to 0.8% (Kiekens et al., 2018). This difference in reported prevalence rates suggests that the degree of psychological distress and/or impairment associated with NSSI can vary substantially across university students, with only a small proportion of that population showing sufficiently severe symptoms to meet *DSM-5* criteria.

#### Monkey Self-Injury

Prevalence of SIB in rhesus monkeys and other closely related macaque species can be established both by examining colony health records to identify monkeys who received veterinary care for self-inflicted wounds and by conducting behavioral surveys of the monkeys in the research colony. Early estimates of the prevalence of SIB derived from a review of colony health records at three major primate facilities revealed a prevalence rate ranging from 11 to 15% of the total research population (Bayne et al., 1995; Bellanca and Crockett, 2002; Lutz et al., 2003b). Assessments were based on the time period the animals were housed in the facility, which is the closest approximation to a lifetime prevalence rate that can be obtained under these conditions. At the specific facility where our intensive SIB research was being conducted, we determined a “lifetime” prevalence rate of 11% (Lutz et al., 2003b). In terms of incidence, monkeys at this facility averaged about 2 major wounding events in a 5-year period (Novak, 2003). Although at first glance this seems like a very low incidence rate, regular behavioral observations of the animals provided a different picture of this disorder. The monkeys actually engaged in self-biting behavior on a daily basis, but serious wounding events requiring veterinary care were infrequent. Instead, these daily biting episodes produced, at most, small cuts or minor skin abrasions (Novak, 2003). Over the years, significant changes in colony management have reduced the “lifetime” prevalence of SIB in primate facilities to a range of 1–5% (Lee et al., 2015).

### Distribution Across Age and Sex

#### Human Self-Injury

Non-suicidal self-injury typically arises in adolescence, with early onset yielding greater vulnerability. Individuals younger than 12 years of age at the time of onset tend to develop more severe forms of NSSI and require more hospital visits (Ammerman et al., 2018). Whether a gender bias exists in NSSI is unclear. In some reports, women appear more vulnerable to this disorder than men (e.g., Favazza and Conterio, 1989), but in others, no gender difference was detected (e.g., Klonsky et al., 2003). A more recent meta-analysis supports the view that NSSI is more common in women than in men, although the difference is greater in clinical populations compared to college/community samples (Bresin and Schoenleber, 2015).

#### Monkey Self-Injury

Similar to NSSI, SIB in rhesus monkeys appears to arise in adolescence (Lutz et al., 2007). However, unlike the gender difference reported for NSSI, initial findings suggested that SIB was much more common in males than in females (Lutz et al., 2003b). More recent surveys of colony health records failed to detect any sex difference (Peterson et al., 2017), but there may be a male bias in severity, perhaps explained by the presence of enlarged canine teeth in males.

### Similarities and Differences in Human NSSI and Rhesus Monkey SIB

The information presented above suggests substantial concordances between human NSSI (as seen in non-clinical populations) and the model of rhesus monkey SIB. The first key similarity is that the form of self-harm (e.g., biting, hitting, head banging, skin gouging, etc.) is highly congruent across humans and monkeys, excluding cutting and burning for which there is no monkey equivalent. Second, both disorders arise spontaneously, without the need for induction. Third, both disorders typically emerge in adolescence, suggesting that certain developmental events play a role in the emergence of both NSSI and SIB. Finally, there is a continuum of severity ranging from milder cases to the more severe forms of wounding, requiring extensive veterinary care in monkeys and hospitalization in humans. Some disparities also exist. Gender differences are not the same. Whereas women are at somewhat higher risk in developing NSSI than men, recent research on monkeys suggests either a slight male bias or no difference between males and females. Additionally, prevalence rates of self-injury are generally lower in monkeys than in humans except when *DSM-5* criteria are applied. Note, however, that the gradual identification of risk factors for SIB (described below) has been used by primate facilities to reduce its prevalence, whereas prevention strategies have yet to be developed for people who are at risk for NSSI (Brown and Plener, 2017). In the remainder of this paper, we will provide more details about our specific model of rhesus monkey SIB, and we will discuss how information about the etiology of the disorder, its biological underpinnings, and potential treatments may inform the study and treatment of NSSI in humans.

## The Model of Self-Injury in Rhesus Monkeys

### Approach

This model focuses on the spontaneous occurrence of SIB in rhesus monkeys in contrast to animal models in which the abnormal behavior is induced experimentally by genetic manipulations or drug treatments (e.g., amphetamine). The consequence of studying spontaneously occurring disorders is that strict causality cannot be evaluated. Instead, this model is based on the identification of risk factors and correlates. Risk factors are those features that increase the probability of a monkey’s chance of developing SIB. By definition, such factors must have predictive value and be present before the disorder appeared, as determined by historical data (Offord and Kraemer, 2000). Some risk factors are fixed (e.g., genotype, rearing practice) whereas others are variable (e.g., length of individual cage housing). In contrast, correlated features are concomitant with the disorder, determined through observations of monkeys with SIB in comparison to healthy controls. In the latter case, the sequential relationship between these features and SIB cannot be determined.

We employed two strategies to understand the etiology of SIB in rhesus monkeys. First, risk factors were identified by examining the colony health and management records and comparing monkeys having a veterinary record of SIB (i.e., at least one self-inflicted wounding episode that required veterinary attention) to monkeys that had no such record. The data set consisted of 362 animals with similar housing. Relevant information in the records included early rearing practice, housing history, health assessments, number of relocations, and number of veterinary procedures (e.g., blood draws). Variables were created from the information and statistically analyzed to identify risk factors for SIB. A similar procedure was carried out at other primate facilities, and commonalities across facilities were noted. Additionally, a subset of monkeys with and without SIB was genotyped for specific genetic polymorphisms previously related to various mental health conditions.

In the second strategy, the same subset of monkeys both with and without SIB were observed and examined intensively to determine the presence of behavioral or physiological features associated with SIB. This approach enabled us to show that the disorder in rhesus monkeys is a syndrome consisting of multiple abnormalities, not just self-injury.

### Features of the Model

We hypothesize that SIB emerges in some monkeys from an interaction between adverse early experience and a genetic predisposition, leading to a dysregulation of the neuroendocrine stress response (Tiefenbacher et al., 2004). Although the mechanism underlying such dysregulation is not yet clear, one intriguing possibility involves epigenetic modifications of glucocorticoid receptor gene expression (Matthews and McGowan, 2019). These factors interact to sensitize the animals to additional stress exposure and subsequently lead to disturbances in other neurobiological systems that manifest as increased aggressiveness (Lutz et al., 2003a), and sleep disruption (Stanwicks et al., 2017). The development of SIB is then viewed as a coping response to ongoing stress exposure.

### Risk Factors: Early Life Stress, Neuroendocrine Stress Dysregulation, and Genetic Polymorphisms

#### Colony Record Data and Links to Early Life Stress

A statistical analysis of the colony health and management records of large populations of monkeys at different primate centers revealed the importance of various life stressors in determining the risk for developing SIB. The most significant risk factor for this disorder was maternal separation of infants at birth followed by nursery rearing. In one study focusing on this specific factor, the ratio of nursery-reared monkeys to maternally reared monkeys that developed SIB was 14:1 (Lutz et al., 2007). The adverse consequences of nursery rearing have also been reported at four other primate centers (Bellanca and Crockett, 2002; Lutz et al., 2003b, 2007; Rommeck et al., 2009; Gottlieb et al., 2013). However, even mother-reared monkeys developed SIB if they experienced stressful events as juveniles. One such stressful event was going onto a research protocol that entailed separation from the social group during this crucial developmental period. Thus, monkeys that developed SIB were separated as juveniles (average age of 14 months) whereas monkeys that remained healthy were separated as adolescents (average age of 36 months) (Lutz et al., 2003b). Finally, some monkeys developed SIB in adulthood which was linked to cumulative stress exposure. The odds of developing SIB in adult monkeys increased with the number of veterinary procedures requiring restraint and sedation (e.g., blood draws) and with the length of time monkeys were individually housed for research purposes (Lutz et al., 2003b; Gottlieb et al., 2013).

#### Neuroendocrine Stress Mechanisms

##### Features of the HPA axis

The hypothalamic-pituitary-adrenocortical (HPA) axis plays a significant role in both the response and recovery to acute stressors. However, when stressors are chronic or cumulative, HPA axis function may become dysregulated. Most studies measure cortisol, the principal output of the HPA axis in humans and non-human primates. Cortisol levels can be measured in many different bodily fluids and tissues, including blood, urine, saliva, feces, sweat, hair, and fingernails. As noted in Table 1, these sample matrices differ with respect to the time frame they represent, the cortisol fraction being measured, units of measure, and susceptibility to being influenced by circadian rhythms (Novak et al., 2013; Meyer and Novak, 2021).

**TABLE 1:** Characteristics of various cortisol sampling matrices.

Matrix	Time Frame	Cortisol Fraction	Unit of Measure*	Subject to Circadian Variability
Blood (plasma or serum)	Minutes	Total (free and bound)	Mass per unit volume	Yes
Saliva	Minutes	Free	Mass per unit volume	Yes
Urine	Hours/Day	Free + (creatinine correction for urine volume)	Mass per unit volume	Yes (unless 24 h urine collection)
Feces	Hours/Day	Free	Mass per unit weight	Yes (unless 24 h fecal collection)
Hair	Months	Free	Mass per unit weight	No
Fingernails	Months	Free	Mass per unit weight	No

*Refers to how sample cortisol concentration is expressed (e.g., ng/ml for plasma cortisol versus pg/mg for hair cortisol).

The selection of sampling matrix depends on the objective of the study. Thus, blood and saliva samples are most appropriate for assessing the time course of HPA responses to acute stressors, whereas hair and fingernails are more relevant for measuring the impact of long term stressors. It should also be noted that the relative concentrations within each matrix are very different. For example, plasma samples contain total cortisol and are many times higher than salivary cortisol samples, which reflect only the free (unbound) cortisol fraction.

##### Role of the HPA axis in SIB

We hypothesized that the stress of early maternal separation was a key factor in the increased vulnerability to SIB in nursery-reared compared to mother-reared monkeys. If this hypothesis was correct, then nursery-reared infant monkeys might show altered HPA axis activity compared to a mother-reared infant group. In two separate studies, nursery-reared monkeys showed lower plasma cortisol concentrations in response to the acute stress, involving a brief separation from their social group for blood sampling and temperament testing, compared to maternally reared monkeys who underwent the same procedures (Capitanio et al., 2005; Kinnally et al., 2008). The blunted stress response in nursery-reared infant monkeys was also present in juveniles with early life stress exposure (Feng et al., 2011). Because this blunted cortisol response initially occurred in infants prior to the typical age of SIB onset, we consider dysregulated HPA axis activity to be a risk factor for this condition.

Studies of adult monkeys have lent additional support for this view. Compared to controls, monkeys with SIB also showed a blunted plasma cortisol response to the acute stress of capture and blood sample collection (Tiefenbacher et al., 2000, 2004). Additionally, plasma cortisol concentrations were negatively correlated with biting rate and with recency of a serious wounding event. In contrast to this blunting effect, chronically *elevated* HPA activity, assessed by cortisol accumulation in hair samples, was observed in SIB monkeys compared to healthy controls (Davenport et al., 2008). These findings are best explained by the presence of persistent long-term HPA activation leading to a compensatory dampening of the normal cortisol rise in response to an acute stressor (Herman et al., 2016). In this form of HPA axis dysregulation, the organism is unable to mount an adequate response to short-term stressful events.

#### Role of Genetics in Stress Dysregulation and SIB

For many years, genetic contributions to complex human behaviors in general and psychiatric disorders in particular were examined using a candidate gene approach in which genes considered likely to be associated with the behavior or disorder were selected *a priori* for study. Because of the limitations of this approach, which include the obvious selection bias, the candidate gene approach has largely been replaced with GWAS. To our knowledge, SIB in monkeys has not yet been investigated using the GWAS approach; however, several earlier studies were conducted relating specific genetic polymorphisms to SIB and to HPA axis function. We reasoned that because most monkeys subjected to nursery rearing (an early life stress) did not develop SIB, the animals that did show the behavioral pathology may have possessed one or gene alleles that conferred vulnerability. To test this hypothesis, a smaller subset of monkeys with and without SIB was genotyped for the presence of several genetic polymorphisms previously associated with various psychiatric conditions. We specifically investigated the following three genetic polymorphisms: the serotonin transporter gene-linked polymorphic region (5-HTTLPR), a single nucleotide polymorphism in the mu-opioid receptor gene (C77G), and regulatory polymorphisms of the tryptophan hydroxylase-2 (TPH2) gene. Only the latter two polymorphic variants were associated with the features of SIB. The mu-opioid receptor variant (G77-containing alleles) was associated with higher beta-endorphin affinity, lower blood cortisol levels in response to an acute stressor, and more aggressive threat behavior in rhesus monkeys (Miller et al., 2004). Additionally, the distribution of rhTPH2 5′-FR haplotypes differed significantly between monkeys with and without SIB, and this genetic difference was also associated with lower plasma cortisol concentrations in response to acute stress exposure (Chen et al., 2010). Although these findings are consistent with the general theory that spontaneously occurring SIB in monkeys likely involves gene by environment interactions, the specific associations discussed here must be considered preliminary given the small sample sizes and lack of replication to date. GWAS based on a larger population of monkeys with and without SIB is needed if we are to advance our understanding of the role of genetics in this disorder.

### Behavioral Correlates of SIB

As mentioned earlier, we hypothesize that spontaneously occurring SIB in rhesus monkeys is one component of a broader constellation of behavioral and neurobiological abnormalities. To test this hypothesis at the behavioral level, we compared SIB and non-SIB monkeys with respect to differences in anxious behavior, social behaviors, and sleep.

#### Anxious Behavior

Typical forms of anxious behavior in macaque monkeys include yawning, scratching, vigilance, and displacement activity. Baseline levels of these behaviors in the home cage did not vary across monkeys with and without SIB. However, anxious behavior can also be assessed with various experimental tests, one of which is the Human Intruder Test (HIT). In this test, an unfamiliar human approaches a monkey, stands in profile, turns to gaze at the monkey, then turns her back, and then finally exits the room. Anxious behavior includes moving away or looking away from the intruder and showing fear responses, especially during the gaze portion of the test. Consistent with the view that heightened anxiety was not a key feature of this disorder, monkeys with SIB showed *lower* levels of anxious behavior during the HIT compared to controls (Peterson et al., 2017). Anxious behavior has been associated with reduced serotonergic activity and with the serotonin transporter gene (5-HTTLPR) short allele. Serotonergic activity in monkeys with and without SIB was evaluated by measuring the concentrations of the serotonin metabolite 5-hydroxyindoleacetic acid in cerebrospinal fluid, by determining the prevalence of the 5-HTTLPR short allele, and by evaluating the serotonergic response to a fenfluramine challenge test. No relationship to SIB was detected in any of these measures, indicating that if serotonin plays a role in the disorder as suggested by the TPH2 gene results, functional dysregulation of the serotonergic system has not yet been identified (Tiefenbacher et al., 2000, 2003; Tiefenbacher S. et al., 2005). Because of this negative outcome, we did not evaluate anxious behavior in humans with NSSI.

#### Deficits in Social Interaction

Although not abnormally anxious, monkeys with SIB showed deficits in social interactions with other monkeys as manifested by high levels of aggressive behavior and low levels of affiliative behavior compared to monkeys without SIB. Biting and aggressive behavior were linked in that 5-min observational samples containing biting episodes had a higher proportion of threats to other nearby monkeys than non-biting samples (Novak, 2003). The act of biting is a species-typical behavior, since monkeys typically bite and wound other monkeys in aggressive altercations. Thus, our findings raised the general question, were self-directed bites a form of redirection to oneself when physical contact with others was prevented?

Threatening behavior was subsequently manipulated by exposing monkeys with and without SIB to an unfamiliar monkey in a different pen. Although the manipulation increased threatening behavior in monkeys with SIB, it did not alter their biting rates (Lutz et al., 2003a). These findings indicate that SIB in rhesus monkeys is not merely redirected aggressive behavior. Thus, the functional relationship between aggression and biting behavior in monkeys requires further study.

#### Sleep Disruption

In early studies of nighttime activity, monkeys with SIB spent more time awake at night than controls (Davenport et al., 2008). These findings were later replicated in a different population of monkeys with and without SIB using infrared surveillance software that could reveal the timing of this activity as it occurred across the night. Using this approach, we found that monkeys with SIB compared to controls showed delayed sleep onset, higher overall activity at night, and longer bouts of activity when awake (Stanwicks et al., 2017). Although these activity data are not definitive with respect to sleep since there was no monitoring of cerebral EEG, the results strongly suggest that several aspects of the sleep cycle (e.g., greater latency to sleep onset and less overall sleep) are abnormal in monkeys with SIB.

## The Rhesus Monkey Model: Parallels Across Risk Factors and Correlates in Humans With NSSI

One of the limitations of our model is that, unlike humans, monkeys cannot directly reveal their feelings or thoughts. Thus, some of the very important correlates of NSSI such as negative affect, dissociative states, and other internalizing symptoms are obviously beyond the scope of this model. Our etiological model of rhesus monkey SIB emphasizes the importance of early life stress combined with genetic vulnerability and HPA axis dysregulation to evoke SIB and other associated features, such as deficits in social interaction and sleep disruption.

A number of human etiological models have been developed to explain how various factors may interact to produce NSSI. In one of these models, Nock (2009) has argued that early life stressors (particularly child abuse) interact with genetic risk factors for deficient cognitive processing and abnormal emotional reactivity. This interaction is then hypothesized to lead to emotion dysregulation and inadequacies in communication, thereby increasing vulnerability to NSSI. In the following section, we consider the extent to which the factors thought to underlie NSSI in humans parallel the risk factors and correlates of SIB in our rhesus monkey model.

### Risk Factors for NSSI

### Early Life Stress

As in the monkey model, early life stress was a significant predictor of NSSI within both community (Yates et al., 2008; Taliaferro et al., 2012) and clinical samples (Kaess et al., 2013). This connection was further supported by a recent systematic review of 20 cross-sectional studies (Serafini et al., 2017). In nearly all of these studies, the development of NSSI was linked to some form of early childhood adversity.

As noted in the monkey model, the impact of stress was not limited to childhood. In females, the risk of developing NSSI increased with each additional stressful event after a baseline of two such events (Steinhoff et al., 2020). NSSI can also develop in adults after exposure to a single highly stressful event. Of the men and women who experienced military sexual assault, over 25% engaged in NSSI; and within that population, the majority indicated that their first act of NSSI occurred after the traumatic event (Holliday et al., 2018).

#### Neuroendocrine Stress Response

There is now growing evidence that HPA axis activity is dysregulated in individuals with NSSI. In these studies, blood or salivary cortisol has typically been measured in response to an acute stressor; only occasionally has hair cortisol been assessed to evaluate chronic stress exposure. As observed in rhesus monkeys, individuals with NSSI showed a blunted response to an acute stressor. In the most recent study, Reichl et al. (2019) compared adolescents engaging in NSSI with their siblings. Adolescents with NSSI reported more childhood adversity and showed a blunted salivary cortisol response to a trauma interview in which they were asked to relive traumatic events. The authors also assessed chronic stress exposure and found hair cortisol concentrations to be elevated in the NSSI group when the data were controlled for smoking behavior. Two other acute stressors have been tested for their effects on salivary cortisol. Individuals with NSSI showed a blunted salivary cortisol response to the Trier Stress Test (Kaess et al., 2012; Klimes-Dougan et al., 2019) but elevated salivary cortisol in response to the cold pressor task (Koenig et al., 2017). It is noteworthy that this elevated cortisol response occurred to a non-psychosocial stressor, whereas the previously mentioned studies that reported blunted cortisol responses used psychosocial stress challenges. Finally, other features of HPA axis dysregulation in people with NSSI have been shown by elevated salivary cortisol concentrations 30 min after awakening (i.e., cortisol awakening response) (Reichl et al., 2016) and a substantially greater suppression of blood cortisol concentrations in response to a dexamethasone suppression test (Beauchaine et al., 2015).

#### Role of Genetics in NSSI

The study of genetic involvement in NSSI suffers from the same limitations as discussed earlier for monkey SIB. A number of reports of GWAS in relation to suicidality have been published; however, to date the only application of this approach to NSSI is the recent study by Campos et al. (2020) that looked for genes related to self-harm behavior and/or self-harm ideation. Although several genes were significantly associated with one or both of these variables, the one with the strongest association with both was *dcc* (deleted in colorectal cancer), a gene known to play an important role in the development of the prefrontal cortex (Manitt et al., 2013). Much more research is needed to understand how genetic variation is related to NSSI versus suicidality, especially since self-harm ideation and behavior leads to suicide attempts in some individuals.

Considering the genetic polymorphism studies that have been associated with monkey SIB or its correlated features, it seems reasonable to mention any prior studies of NSSI involving the same genes to determine if parallel findings were obtained. Our literature search revealed that only one of the genes associated with SIB has also been studied with respect to NSSI, namely the TPH2 gene. In this case, however, the genetic variants have been linked specifically to the development of suicide risk, not to NSSI (see meta-analysis by Ottenhof et al., 2018). Nevertheless, this finding is of interest given the substantial evidence that NSSI is a gateway to suicide (Griep and MacKinnon, 2020). Also related to the serotonergic system is a study of the serotonin transporter gene length polymorphism (5-HTTLPR) in NSSI. The polymorphic variants consist of a short and a long allele, of which the short allele is associated with lower levels of serotonergic activity. Hankin et al. (2015) found that individuals who carried the short allele and experienced chronic interpersonal stress showed high levels of NSSI. Consistent with the genetic results is a report of lower plasma serotonin concentrations in a community sample of self-injuring adolescents compared to controls (Crowell et al., 2008). Although we found no association between 5-HTTLPR and SIB in monkeys, it remains possible that this was because of the small sample size or other mitigating factors.

### Correlates of NSSI

In our monkey model, SIB was significantly associated with deficits in social communication, manifested primarily as aggressive and threatening behavior, and sleep disruption. In general, NSSI has been associated with many different features, the presence of which varies considerably across individuals. Below, we assess the two primary correlates identified in monkey SIB and determine their potential role in NSSI.

#### Aggressive Behavior

An extensive literature supports an association of NSSI with other-directed aggression both in high school students (Brunner et al., 2007) and clinical populations (Boxer, 2010). The association between aggressive behavior and NSSI has been shown to persist in adolescents and university students even when controlling for the effects of emotion regulation (Tang et al., 2013; Sorgi et al., 2020). One form of aggression in teenagers is bullying behavior, and NSSI is associated with frequent victimization (Fisher et al., 2012; Jantzer et al., 2015). However, it has also been linked to perpetrators of bullying (Esposito et al., 2019). Stress-related disorders appear to mediate the relationship between aggression and NSSI in some individuals. Veterans with PTSD who also engaged in NSSI were more likely to threaten and commit violence against others than veterans with PTSD but without NSSI (Calhoun et al., 2017).

Is NSSI in aggressive individuals a form of aggression toward self? This intriguing idea is supported by the finding that aggressive individuals tend to engage in specific forms of NSSI, namely hitting and punching oneself. Kleiman et al. (2015) found that trait aggression was associated both with the form of NSSI expression and with a lifetime history of self-hitting behavior. Hitting does not have to be directed to the self to be injurious. Wall/object punching was common among war veterans diagnosed with PTSD (43%). This behavior resulted in injuries and yielded the typical post-injury relief associated with the commission of other forms of NSSI (Kimbrel et al., 2017).

#### Sleep Disruption

Self-injurious behavior in rhesus monkeys was associated with significant sleep disturbances including delayed sleep onset, shorter sleep length, and more awakenings per night. Parallel to the monkey model, recent studies have suggested a strong relationship between NSSI and sleep problems. Nearly all of these studies rely on self reports of sleep quality using various scales. They do not measure sleep directly with polysomnography or examine nighttime activity patterns using actigraphy or surveillance software. Regardless of this limitation, virtually all of these studies revealed a pattern of disrupted sleep. The most common forms of dysregulated sleep endorsed by individuals with NSSI were insomnia, nightmares, and poor sleep quality. Insomnia was significantly associated with NSSI in a community sample of adolescents, an effect that persisted after controlling for depressive symptoms (Latina et al., 2021), and also in a survey of Norwegian adolescents (Hysing et al., 2015). Insomnia symptoms were additionally linked to the recency of NSSI (Bandel and Brausch, 2020). In some cases, insomnia was influenced by mental health conditions and/or demographic factors and was not independently associated with NSSI (see Ennis et al., 2017 for a university sample; see Liu et al., 2017 for an adolescent sample). However, in these same two studies, NSSI was independently associated with a different form of sleep disruption, namely nightmares. Nightmares were also a major form of sleep disruption endorsed by the parents of young children with NSSI (Singareddy et al., 2013). Poor sleep quality, without specific reference to the forms of sleep disturbance, has additionally been linked with NSSI (Liu et al., 2017; Asarnow et al., 2020). In the one study to date in which sleep was monitored by polysomnography, only one variable differentiated children with and without NSSI. The percentage of time spent in REM sleep was greater in the NSSI group (Singareddy et al., 2013).

The findings described above demonstrate that aggression and sleep disruption, two behavioral features present in monkeys with SIB, are also frequently found in people with NSSI. These parallels suggest that the common risk factors for SIB in monkeys and NSSI in humans may work through closely related neurobiological pathways to yield similar multi-faceted behavioral syndromes.

## Function: What Purpose Does Self-Injury Serve?

### Rhesus Monkey SIB and the Environment

One of the more perplexing challenges has been to understand the why of monkey SIB. Why do monkeys harm themselves and why does this deviant behavior continue to persist in individual monkeys despite intervention attempts? From a broad functional perspective, the commonly held view is that animals engage in abnormal behavior because of powerful reinforcement contingencies. A number of functional models have been proposed to explain what those reinforcement contingencies might be.

In monkeys, we examine how SIB might be used to mitigate the effects of adverse environments. Two hypotheses suggest that SIB serves to ameliorate the ongoing and long lasting effects of adverse environments. Both focus on the extreme ends of a continuum of environmental quality ranging from environments that are under-stimulating and impoverished to those that are over-stimulating and chaotic. Superimposed on this continuum is the presence of wide individual differences in reactions to environments, which poses a barrier to effective treatment.

#### Impoverished Environments and Sensation Seeking

According to this hypothesis, SIB provides a form of sensory input to an animal existing in a barren environment with little stimulation. As a corollary, SIB is a manifestation of a species-typical response, namely threatening/aggressive behavior, which is thwarted in the absence of social partners and instead redirected to the self. Thus, SIB is positively reinforcing by providing increased stimulation and allowing for the expression of aggressive behavior. If this hypothesis is true, then changing the environment by addressing sensory-perceptual needs and providing adequate social partners should lead to a reduction in SIB. Currently, there is some support for this hypothesis. Providing social partners resulted in a reduction in SIB in some monkeys that previously had been housed in individual cages because of the requirements of specific research projects (Weed et al., 2003; Fontenot et al., 2006). In contrast, however, enhancing the inanimate features of the sensory-perceptual environment was not a particularly effective strategy. Biting behavior rates were unaffected by the addition of manipulable objects and foraging devices (Rommeck et al., 2009), puzzle feeders (Novak et al., 1998), or play cages (Griffis et al., 2013). To put these latter findings in perspective, it is likely that strong individual differences in preferences for various environmental enhancements may have obscured the effectiveness of these changes on reducing SIB.

#### Stressful Environments and Arousal Reduction

This hypothesis posits that monkeys bite themselves to reduce their arousal in response to stressful stimuli or, as a corollary, to focus their actions inward as a way of avoiding the stressors in their environment. In this scenario, SIB is maintained through a process of negative reinforcement. Some limited support for this view comes from the finding that heart rate patterns in 10 monkeys with SIB were elevated just prior to a biting episode and returned to baseline shortly thereafter (Novak, 2003). A more general test of this hypothesis requires identifying the stressors to which individual animals might react, keeping in mind wide individual variation, and then developing a strategy to mitigate that stress exposure and observing corresponding reductions in SIB.

One of the more effective ways to reduce the stressfulness of various research and veterinary procedures is through familiarization and positive reinforcement training. Monkeys have been trained to give blood samples by freely extending their legs (Graham et al., 2012) and provide saliva samples by chewing on dental rope (Lutz et al., 2000). Additionally, positive reinforcement training has been used to acclimate monkeys to transport boxes and restraint devices (Mason et al., 2019). At the present time, there are no studies assessing the effects of such training on SIB; but there is strong evidence that training is associated with a reduction in other forms of abnormal behavior in monkeys such as repetitive stereotyped behaviors (Baker et al., 2009; Coleman and Maier, 2010).

There are two limitations to the idea that SIB can reduce the effects of adverse environments. First, environmental quality is a construct and does not take into account the dynamic temporal fluctuation in environmental stimulation that occurs in any environment. Additionally, it is likely that motivations for SIB vary both across individuals and within an individual over time.

### Human NSSI

Many different theoretical models have been proposed to explain why humans injure themselves. There is evidence for the involvement of the opioid system and for the role of the environment broadly defined. And as noted with the rhesus monkeys, some of these models posit that NSSI is a form of positive reinforcement and others link NSSI to negative reinforcement. More importantly, new advances now allow scientists to obtain information that bears directly on the act of self-injury. Using ambulatory assessment, self-report information as well as behavioral and physiological measures can be obtained from the individuals in real time immediately prior to and directly after episodes of NSSI (Trull and Ebner-Priemer, 2013).

#### Environmental Factors

A variety of factors elicited through an individual’s interaction with their environment can provoke episodes of NSSI. As described in self reports, these episodes are reinforcing, at least temporarily, leading to an ongoing cycle of NSSI-elicited relief, followed by reinstatement of the original problem, leading to more bouts of NSSI. The four function model proposed by Nock and Prinstein (2004) organizes these factors along two dimensions: intrapersonal (e.g., emotion regulation) vs. interpersonal (e.g., social interaction), and negative vs. positive reinforcement. At an intrapersonal level, NSSI may produce negative reinforcement by reducing a negative internal state (e.g., anxiety) or produce positive reinforcement by increasing stimulation in a monotonous environment (Nederkoorn et al., 2016). At the interpersonal level, NSSI may be negatively reinforcing by reducing unwanted interactions with others, or conversely it may be positively reinforcing by increasing attention to the individual and enhancing social support.

The four function model has been used successfully to classify the forms of NSSI committed by incarcerated populations (Power et al., 2016) and by Swedish community members (Zetterqvist et al., 2013). However, in one study, intrapersonal functions of NSSI were emphasized more and were more likely to produce relief than interpersonal functions (Brausch and Muehlenkamp, 2018). Moreover, a systematic review of 35 ambulatory assessment and diary studies provided support for only the negative intrapersonal function (Taylor et al., 2018; Hepp et al., 2020). These differing results may not be surprising given that the relative importance of these four functions most likely differs between individuals and within individuals over time. It does suggest that a major reason for engaging in NSSI is to reduce negative states of various kinds as has been proposed in the Experiential Avoidance Model (Chapman et al., 2006) and the Emotional Cascade Model (Selby et al., 2013).

The parallels between the monkey and human data are less clear with respect to how an interaction with the environment elicits self-injury. The strongest potential alignment between the monkey and human data comes from the view that in humans, NSSI can reduce adverse mental states triggered by external situations, and in monkeys, SIB may reduce heightened arousal caused by stressful environmental events. One significant advantage in the monkey studies is the ability to redesign environments to mitigate stress exposure and to assess the possible impacts on SIB.

## Resolving Self-Injury

Considerable effort has been expended in developing effective treatments for self-injury in both human and non-human primates. In monkeys, this effort involves environmental redesign and stress reduction procedures (as mentioned in the section under function) as well as pharmacotherapy. In fact, one of the significant advantages of the monkey model is in evaluating the efficacy of new treatments, whether it be environmental, pharmacological, or some combination, in overcoming SIB, prior to their implementation in humans.

### Pharmacological Treatments for SIB

Pharmacotherapy is considered a treatment of last resort for non-human primates and is primarily used to treat serious instances of SIB that result in repetitive wounding episodes. Although pharmacotherapy has efficacy in reducing SIB, there are potential risks to health and well-being. These risks include side effects, such as sedation and short-term gastrointestinal distress, development of tolerance requiring escalation of dosing schedules, withdrawal symptoms on dose reduction, and relapse once the drug has been removed. The ideal drug should have three properties: (1) the side effects should be short lasting, (2) the drug should induce changes in brain neurochemistry leading to a major reduction if not a cessation of SIB, and (3) the neurochemical changes and therapeutic benefits should persist after the treatment ends. Over the past 20 years, drugs that alter the activity of the GABAergic, serotonergic, adrenergic, or opioidergic neurotransmitter systems have been tested for their efficacy in reducing SIB in monkeys. To date, none of the tested compounds fits the ideal therapeutic profile, but some drugs come closer than others. In the following section we review each of the systems mentioned and associated drug therapies in order from least effective to most effective.

#### GABAergic System

Historically, the GABA_*A*_ receptor-positive allosteric modulator, diazepam, was used to treat SIB in monkeys, based on its anxiolytic properties. However, subsequent evaluations revealed marked individual differences in response to this drug. For example, when administered to eight monkeys with SIB, only four showed a reduction in wounding rates; the remaining monkeys actually got worse (Tiefenbacher S. T. et al., 2005). Because of this disparate reaction, diazepam is no longer recommended for alleviating SIB in rhesus monkeys.

#### Serotonergic System

Selective serotonin reuptake inhibitors (SSRIs) have also been used to manage episodes of SIB in rhesus monkeys. Administration of the SSRI fluoxetine and the 5-HT1A receptor partial agonist, buspirone, led to a 50% reduction of SIB during the early weeks of treatment (Fontenot et al., 2005). The post-treatment period was too short (2 weeks) to assess relapse. In a second study, fluoxetine was more effective than venlafaxine (a combined serotonin and norepinephrine reuptake inhibitor; SNRI) in managing SIB. This study only evaluated dosing strategies and did not follow the monkeys post treatment (Fontenot et al., 2009). Serotonergic activity can also be manipulated by adding the precursor L-tryptophan to the diet. In rhesus monkeys, SIB decreased during L-tryptophan administration, but the monkeys relapsed once the dietary supplementation ended (Weld et al., 1998).

#### Adrenergic System

Guanfacine, an α2A adrenergic receptor agonist, was used to treat SIB in two macaques and one baboon. Self-biting behavior was eliminated by administration of guanfacine; however, the three animals eventually relapsed after the treatment ended (Macy et al., 2000). A wound scoring scale was used in a second study to evaluate the effect of guanfacine on self-inflicted wounds in rhesus monkeys (Freeman et al., 2015). Treatment reduced the severity of inflicted wounds and this effect persisted into a post-treatment period for about 100 days before relapse occurred in some monkeys.

#### Opioidergic System

To date, the most promising treatment for SIB is the long-acting opioid receptor antagonist naltrexone because it affects not only SIB but also some correlated brain anomalies. Administration of naltrexone reduced SIB during treatment and had long lasting benefits that were still present 110–200 days post treatment (Kempf et al., 2012). In a follow-up study, the postmortem brains of SIB monkeys treated with naltrexone, untreated SIB monkeys, and healthy controls were examined. The results revealed significant immune activation and atrophy of both gray and white matter astrocytes in untreated SIB monkeys compared to healthy controls. Moreover, these differences were abolished in monkeys that received naltrexone treatment (Lee et al., 2015). Although the sample sizes were relatively small in the two studies, taken together they are consistent with the hypothesis of opioidergic system involvement in SIB in rhesus monkeys. While on board, naltrexone presumably blocked the reinforcing effects of self-biting-induced opioid release, leading to an extinction of the behavior. The mechanism by which this extinction continued even after the end of treatment is unknown; however, the neuropathological findings suggest that a reversal of CNS immune activation and astrocyte atrophy may have played some role in this effect.

### Pharmacological and Psychotherapeutic Treatments for NSSI

#### Pharmacological Approaches

Typically, psychotherapeutic approaches are more commonly implemented in the treatment of NSSI than pharmacological approaches. In part, this difference may be related to the potential risks to health and well-being associated with the adverse reactions of some individuals to drug treatments developed for mood and stress-related disorders. As in rhesus monkeys, these reactions can include side effects such as sedation, development of tolerance, and withdrawal symptoms upon removal of the treatment. Certain drugs can also increase the risk of suicidal ideation (Zisook et al., 2009), and some individuals may either drop out or show poor compliance in taking their medication. Lastly, individual differences in drug response are difficult to predict and manage, and relapse (a reappearance of NSSI) may occur after the treatment is discontinued.

Despite these concerns, drug treatment may be necessary to treat very severe forms of NSSI. Some empirical evidence exists to support the efficacy of pharmacotherapy in reducing or eliminating NSSI. In common with the monkey studies are the use of SSRIs (fluoxetine), SNRIs (venlafaxine), and opioid antagonists (naltrexone). In addition, two atypical antipsychotics (aripiprazole and ziprasidone) have yielded some efficacy in reducing NSSI. In most cases, pharmacotherapy involved patients with borderline personality disorder. Borderline patients showed a reduction in NSSI with fluoxetine (Markovitz et al., 1991), venlafaxine (Markovitz and Wagner, 1995), and naltrexone (Sonne et al., 1996). In the latter study, patients were treated with naltrexone for only 1 week followed by a 1-week post-treatment period in which all five patients experienced relapse. However, the short period of treatment was probably not sufficient to establish drug efficacy. In another patient population, seven females diagnosed with borderline personality disorder received naltrexone for their clinically significant SIB. Naltrexone eliminated NSSI in six of seven patients during treatment. Two patients, who had discontinued naltrexone, showed a resumption of NSSI, which was eliminated when the naltrexone was reinstated (Roth et al., 1996). In the only randomized clinical trial with borderline patients, the atypical antipsychotic, aripiprazole, led to a cessation of NSSI which persisted for 18 months after treatment in most of the drug treated group compared to the placebo group (Nickel et al., 2006, 2007).

These studies suggest that several different kinds of medications may have acute beneficial effects on NSSI. It is possible that at least in some cases, reductions in self-harm are related more to the ability of the medication to temporarily alleviate the patient’s dysphoric state, rather than targeting the specific mechanisms underlying the behavioral pathology. Most importantly, much more research needs to be done to determine the ability of pharmacotherapeutic interventions to produce lasting reductions in NSSI post-treatment.

#### Psychotherapeutic Approaches

Many different psychotherapeutic approaches have been tried as a treatment for NSSI. A review of all possible psycho-social interventions is outside the scope of this review, particularly because such interventions are difficult to model using non-human primates. We restrict our brief discussion to a recent meta-analysis and a systematic review of randomized control trials of various treatment interventions. A meta-analysis of 25 randomized control trials revealed that most treatments yielded only small or moderate effect sizes when compared to active controls. The most noteworthy approach was dialectical behavior therapy targeted for adolescents (DBT-A) which had moderate effects in reducing NSSI (Kothgassner et al., 2020). In a systematic review, Glenn et al. (2019) noted that currently, there are few Level 1 “well established” interventions for NSSI, with one exception being DBT-A. In both studies, the authors point to the need for independent replication of treatments that currently fall into the category of Level 2 “probably efficacious” in reducing NSSI.

Some of the problems associated with pharmacotherapy as a treatment also pertain to psychotherapy. Individual reactions to psychotherapy can be difficult to predict and may require alteration in therapy protocols. Some individuals may show low compliance with protocols. Furthermore, reinstatement of NSSI may occur once the therapy is discontinued. All of this points to the need for new interventions and prevention strategies, particularly in the case of severe, unremitting NSSI.

## Discussion

In contrast to various genetic, physiological, and infectious diseases, mental health disorders are challenging to model in animals. Many of the symptoms are subjective in nature and are identified primarily through self-report. Additionally, relevant biomarkers that could be modeled in animals are often lacking. Nor are simple diagnostic tests available that can determine whether an individual has or does not have a mental health disorder (Nestler and Hyman, 2010).

Non-suicidal self-injury is somewhat different than other mental health disorders in that its starting point is a group of behavioral acts that yield a discernible physical outcome of tissue damage. That aspect can clearly be modeled in non-human primates, some of whom develop a pathology in which they bite themselves to the point where they require veterinary care. However, the many varied functional explanations provided by individuals for engaging in NSSI, such as overcoming negative emotions, achieving tension relief, or seeking social support, cannot readily be studied in non-human primates because of an inability to communicate their inner states.

Despite the fact that individuals with NSSI can verbalize the subjective consequences of performing acts of self-harm, we still know relatively little about the biological mechanisms that give rise to this disorder. Thus, a major strength of the present monkey model lies in its ability to fill this gap by providing detailed information on the neurobiological and genetic mechanisms underlying SIB, identifying the physiological and behavioral correlates of SIB, and developing novel treatment strategies. These findings have suggested new avenues for investigation in individuals with NSSI, some of which are now being implemented.

The value of any animal model depends on whether it is demonstrably valid with respect to the human condition. Nestler and Hyman (2010) discussed the three major kinds of validity of animal models in biological psychiatry. In face validity, the animal model should mimic the phenotype of the disorder in humans. In this regard, similarities are seen in both the forms and outcomes of self-harm in NSSI and SIB. Furthermore, in both monkeys and humans, the disorder typically arises spontaneously in adolescence, although it can also be triggered in response to stressful events in adults of both species. Construct validity refers to the concordances of biological dysfunctions associated with the etiology of the disorder and its neurobehavioral features. As discussed in this review, NSSI in humans and SIB in rhesus monkeys are commonly associated with early life stress or trauma, and individuals suffering from the respective disorders also show HPA axis dysregulation, abnormal social communication, and sleep disruption. Lastly, predictive validity refers to whether a model can predict the ability of treatments to induce remission. In the present case, this applies to pharmacotherapy, and findings indicate that naltrexone is one of the most effective drug treatments for both NSSI and SIB.

No animal is a perfect substitute for human beings. Despite the close genetic relationship between rhesus monkeys and humans, the two species differ in important ways. Compared to humans, the rhesus monkey cerebral cortex is not as well developed, their higher order cognitive capabilities are much more limited, and they have a different social structure. In addition to these general considerations, there are also specific limitations of the present animal model. One such limitation is its inapplicability to many populations that engage in NSSI. These include individuals with pervasive developmental disabilities or genetic disorders such as Lesch-Nyhan syndrome. Moreover, application of the rhesus monkey model to cases of NSSI comorbid with mood or anxiety disorders is probably incomplete, since the animal model does not attempt to recapitulate symptoms of depressive- or anxiety-like behaviors. These considerations highlight the heterogeneity of NSSI and the virtual impossibility of any single animal model to successfully reproduce all instances of this disorder. We also note that the predictive validity of the model with respect to pharmacotherapy is based almost entirely on drugs used to treat patients with borderline personality disorder. One of the major populations that we have attempted to model, specifically adolescents who engage in self-harm but have not been formerly diagnosed with any other psychiatric disorder, are not often referred for pharmacotherapeutic interventions.

In conclusion, this review has presented an animal model of NSSI that recapitulates many of the features of this behavioral disorder and that reasonably well satisfies the three main kinds of validity used in biological psychiatry. The need for a valid animal model of NSSI is prompted by two important facts. First, statistics show that this disorder is increasing in prevalence among the general population. Second, NSSI is often accompanied by suicidal ideation and, ultimately, by suicide attempts. Consequently, there is an urgent need for the medical community to learn how best to treat individuals with severe NSSI and, preferably, to prevent it from occurring in the first place.

## Author Contributions

MN and JM contributed equally to the conception of the idea, performing the bibliographic search, and drafting the article. Both authors approved the submitted version.

## Conflict of Interest

The authors declare that the research was conducted in the absence of any commercial or financial relationships that could be construed as a potential conflict of interest.

## Publisher’s Note

All claims expressed in this article are solely those of the authors and do not necessarily represent those of their affiliated organizations, or those of the publisher, the editors and the reviewers. Any product that may be evaluated in this article, or claim that may be made by its manufacturer, is not guaranteed or endorsed by the publisher.
